# *qDTY_1.1_*, a major QTL for rice grain yield under reproductive-stage drought stress with a consistent effect in multiple elite genetic backgrounds

**DOI:** 10.1186/1471-2156-12-89

**Published:** 2011-10-18

**Authors:** Prashant Vikram, BP Mallikarjuna Swamy, Shalabh Dixit, Helal Uddin Ahmed, Ma Teresa Sta Cruz, Alok Kumar Singh, Arvind Kumar

**Affiliations:** 1 International Rice Research Institute (IRRI), DAPO Box 7777, Metro Manila, Philippines; 2 V.B.S. Purvanchal University, Jaunpur, India

## Abstract

**Background:**

Drought is one of the most important abiotic stresses causing drastic reductions in yield in rainfed rice environments. The suitability of grain yield (GY) under drought as a selection criterion has been reported in the past few years. Most of the quantitative trait loci (QTLs) for GY under drought in rice reported so far has been in the background of low-yielding susceptible varieties. Such QTLs have not shown a similar effect in multiple high- yielding drought-susceptible varieties, thus limiting their use in marker-assisted selection. Genetic control of GY under reproductive-stage drought stress (RS) in elite genetic backgrounds was studied in three F_3:4 _mapping populations derived from crosses of N22, a drought-tolerant *aus *cultivar, with Swarna, IR64, and MTU1010, three high-yielding popular mega-varieties, with the aim to identify QTLs for GY under RS that show a consistent effect in multiple elite genetic backgrounds. Three populations were phenotyped under RS in the dry seasons (DS) of 2009 and 2010 at IRRI. For genotyping, whole-genome scans for N22/MTU1010 and bulked segregant analysis for N22/Swarna and N22/IR64 were employed using SSR markers.

**Results:**

A major QTL for GY under RS, *qDTY_1.1_*, was identified on rice chromosome 1 flanked by RM11943 and RM431 in all three populations. In combined analysis over two years, *qDTY*_*1.1 *_showed an additive effect of 29.3%, 24.3%, and 16.1% of mean yield in N22/Swarna, N22/IR64, and N22/MTU1010, respectively, under RS. *qDTY*_*1.1 *_also showed a positive effect on GY in non-stress (NS) situations in N22/Swarna, N22/IR64 over both years, and N22/MTU1010 in DS2009.

**Conclusions:**

This is the first reported QTL in rice with a major and consistent effect in multiple elite genetic backgrounds under both RS and NS situations. Consistency of the QTL effect across different genetic backgrounds makes it a suitable candidate for use in marker-assisted breeding.

## Background

Rice is the world's most important food crop and a primary food source for about half of the world's population. The frequent occurrence of abiotic stresses such as drought and submergence has been identified as the key factor for low productivity in the rainfed ecosystem. Rainfed upland and rainfed lowland ecosystems contribute only 21% of the total production from 38% of the cropped area. In rainfed uplands, rainfed shallow lowlands, and rainfed medium lowlands, drought has been the single largest factor for lower yield in rice. Drought during the cropping season directly affects GY, particularly at the reproductive stage, which is the most devastating stage [1,2]. Recent climate change estimates predict the water deficit to further deteriorate in the years to come [3] and the intensity and frequency of drought are predicted to become worse [4]. Worldwide, drought affects approximately 23 million ha of rainfed rice [5]. Among different rainfed regions, eastern India, with around 13.6 million ha of drought-prone area, is the largest in the world [6]. From the beginning of the Green Revolution era in rice in 1965 till 2009, on 14 occasions, rice production in India failed to achieve the estimated production level. Drought caused this lower production on 11 of the 14 occasions [7]. Severe drought witnessed in 2002 and 2009 caused a significant reduction in rice as well as in total food production in India. In 2002, 29% of the geographical area suffered from drought due to 19% annual rainfall deficiency. Compared to the previous year, rice production fell by 21.5 million tons. Similarly, in 2009, total rice production declined by approximately 10.02 million tons [7].

Despite the importance of drought as a constraint, little effort has been devoted to developing drought-tolerant rice cultivars. Most of the high-yielding varieties--IR36, IR64, MTU1010, Swarna, and Samba Mahsuri--grown in rainfed areas are varieties bred for the irrigated ecosystem and they were never selected for drought tolerance. In drought years, these varieties have high yield losses, leading to a significant decline in rice production [8]. In the absence of high-yielding, good-quality drought-tolerant varieties, farmers in the rainfed ecosystem continue to grow these drought-susceptible varieties.

The earlier approach to improve GY under RS through selection based on secondary traits such as root architecture, leaf water potential, panicle water potential, osmotic adjustment, and relative water content [9-12] did not yield the expected results. Breeders and physiologists practiced selection for secondary traits as several earlier studies reported low efficiency of direct selection for GY under RS [13-15]. Similarly, at the molecular level, initial efforts in rice were devoted to mapping QTLs for secondary drought-related traits such as root morphology and osmotic adjustment [16-18]. Marker-assisted selection of QTLs for secondary traits has not been effective for improving GY under RS.

Recent studies at IRRI have shown moderate to high heritability of GY under RS [8,19,20], suggesting direct selection for GY under RS to be a practical approach [8,21]. Further, the feasibility of combining high yield potential with GY under RS has also been reported [8,21]. By employing direct selection for GY under RS, several promising breeding lines for rainfed lowland and rainfed upland have been identified recently [22,23].

Marker-assisted mapping and the introgression of major-effect QTLs for GY under RS could be an efficient and fast-track approach for breeding drought-tolerant rice varieties [19]. For drought, most quantitative trait loci (QTLs) identified in rice and other crops are mostly in the background of non-elite genotypes. A desirable QTL allele discovered in non-elite genetic material and showing a large effect may not offer any improvement in the improved genetic background because the allele may already be ubiquitous in current varieties [24]. In rice, three recent reports indicate that QTLs with large effects on GY under RS may not be uncommon. Bernier et al. [19] reported a QTL on chromosome 12 in a Vandana/Way Rarem population explaining about 51% of the genetic variance for GY under severe upland RS. Kumar et al. [25] reported a major QTL for GY under lowland RS in CT9993/IR62266 population on chromosome 1 explaining 32% of the genetic variance. Venuprasad et al. [26] identified a major QTL for GY under RS on chromosome 3 that explained 36% of the genetic variance.

The lack of repeatability of QTL effects across different populations (QTL × genetic background) and across environments (QTL × environmental interaction) has been the other factor limiting the use of QTLs in molecular breeding [27-30]. Bernier et al. [31] reported stability of *qDTY_12.1 _*across different environments. However, the repeatability of drought yield QTL effects over different genetic backgrounds has not been reported to date in rice. With the prevalence of a few prominent varieties being cultivated on millions of hectares in major drought-prone areas, identifying major QTLs for GY under RS in the background of improved mega-varieties and introgressing the identified QTLs in them could be a suitable marker-assisted breeding (MAB) strategy.

Swarna, IR64, and MTU1010 are high-yielding varieties grown on millions of hectares in irrigated and rainfed ecosystems in India [32]. Swarna and IR64 are highly susceptible to RS [26,33]. MTU1010 is less susceptible than IR64 and Swarna [22]. This study was undertaken with the aim to discover QTLs for GY under RS with a large and consistent effect in the background of high-yielding mega-varieties Swarna, IR64, and MTU1010.

## Methods

### Experimental details

Experiments were conducted at the International Rice Research Institute (IRRI), Los Baños, Laguna, Philippines, in DS2009 and DS2010. IRRI is situated at latitude 14°13'N and longitude 121°15'E. The soil type is a Maahas clay loam, isohyperthermic mixed typic tropudalf. The experiments were sown on December 22 in 2008 and December 20 in 2009. DS drought screening at IRRI has been highly successful due to lesser rainfall and longer dry spells. The performance of genotypes in dry-season screens at IRRI has been reported to be correlated with wet-season screens in India [22].

### Plant material

Nagina 22 (N22), the drought-tolerant donor, is a short-duration (90-95 days) variety developed by selection from landrace Rajbhog from Nepal. Swarna (MTU 7029) is a semi-dwarf high-yielding long-duration (140-145 days) variety developed at the Regional Agricultural Research Station, Maruteru, Andhra Pradesh, India. It is one of the most popular high-yielding cultivars for irrigated and rainfed rice ecosystems in India, Nepal, and Bangladesh [34]. Swarna is highly susceptible to RS [22,26]. IR64 is a well-known semi-dwarf high-yielding medium-duration (112-118 days) variety developed at IRRI. It is grown on large areas in South and Southeast Asia and East Africa. IR64 is also susceptible to RS [33]. MTU1010 is a semi-dwarf high-yielding medium-duration (112-118 days) variety developed at the Regional Agricultural Research Station, Maruteru, Andhra Pradesh, India. It is grown in large areas in central, southern, and eastern India. MTU1010 is moderately susceptible to RS [22].

Three F_3:4 _populations, N22/Swarna, N22/IR64, and N22/MTU1010, were developed. N22 was crossed with Swarna, IR64, and MTU1010 and F_1_s were selfed. Single seeds from each F_2 _plant were selected and bulked. F_3 _seeds were grown and harvested individually. Five hundred plants from each population were harvested. In our study, 292 F_3 _plants in N22/Swarna, 289 in N22/IR64, and 362 in N22/MTU1010 populations were used for mapping large-effect QTLs for GY under RS.

### Phenotyping under RS and NS conditions

Three populations were screened under lowland RS and NS conditions in DS2009 and DS2010. Both RS and NS experiments were laid out with an alpha lattice design in two replications with a 5-meter (m) single-row plot having row spacing of 0.2 m. For both RS and NS trials, seeds were sown in a nursery and 21-day-old seedlings were transplanted. Single seedling per hill was transplanted with 0.15-m spacing between the hills in the row. In NS conditions, 5 cm of standing water was maintained after transplanting throughout the crop season and drained before harvesting. Inorganic fertilizers--nitrogen, phosphorus, and potassium (NPK)--were applied at 120:30:30 kg ha^-1^. To control weeds, insect pests, and snails, appropriate chemical control measures were used. Bayluscide (niclosamide, 0.25 kg a.i. ha^-1^) was sprayed just after transplanting to control snails. To control weeds, Sofit (pretilachlor ± safener, 0.3 kg a.i. ha^-1^), a post-emergence herbicide, was sprayed 4 days after transplanting (DAT). Furadan (carbofuran, 1 kg a.i. ha^-1^) was applied at 5 DAT, followed by Cymbush (cypermethrin, 1 L ha^-1^) ± Dimotrin (cartap hydrochloride, 0.25 kg a.i. ha^-1^) at 16 DAT to control insect pests. RS experiments were sown and maintained like the NS experiments up to 30 DAT. At 30 DAT, water from the RS experiments was drained. Stress was imposed until severe leaf rolling [35] was observed in at least 70% of the lines in each population. At severe stress, life-saving irrigation was provided by flash flooding. The field was drained again 24 hours after irrigation for a second RS cycle to occur [20].

Data on days to 50% flowering (DTF), plant height (PH), biomass (BIO), harvest index (HI), and GY were recorded. DTF was recorded after the exertion of panicles of 50% of the plants of each plot. PH (cm) was recorded as height in centimeters from the soil surface to the tip of the panicle on the main tiller at maturity before harvesting from three random plants and the mean was taken. BIO (g m^-2 ^converted to kg ha^-1^) was harvested from a 1-m linear length in each plot. Biomass samples were then oven-dried, weighed, and threshed for grain weight. Harvest index was calculated using the formula:

$$\text{Harvest index = }\frac{\text{Grain weight}}{\text{Total biomass}}$$

GY (g m^-2 ^converted to kg ha^-1^) under RS and NS situations was harvested from the whole plot at physiological maturity and dried to 12% moisture [19,26].

### Genotyping

#### DNA extraction and amplification of PCR products

For DNA extraction, freeze-dried samples were used. Freeze-dried leaf samples were cut in eppendorf tubes and ground through a GENO grinder. Extraction was carried out by the modified CTAB method [36]. DNA samples were stored in 2-mL deep-well plates (Axygen Scientific, California, USA). DNA samples were quantified on 0.8% agarose gel and concentration adjusted to approximately 25 ng μL^-1^. PCR amplification was done with a 15-μL reaction mixture having 50 ng DNA, 1× PCR buffer, 100 μM dNTPs, 250 μM primers, and 1 unit *Taq *polymerase enzyme. To resolve the PCR products, 8% non-denaturing polyacrylamide gels (PAGE) were used [37]. Parental polymorphism survey was done between N22, Swarna, IR64, and MTU1010 with 682 rice simple sequence repeat (SSR) markers taken from already available rice genetic and sequence maps [38-40].

#### Genotyping of N22/MTU1010 population

Genotyping of N22/MTU1010 population was carried out with 125 polymorphic SSR markers distributed throughout the genome. The map order of the SSR markers used for genotyping was inferred from the Azucaena/IR64 genetic map http://www.gramene.org[41]. For the markers not available in a reference map, order according to physical distance was used [41].

#### Bulk segregant analysis in N22/MTU1010, N22/Swarna, and N22/IR64 populations

Bulked segregant analysis (BSA) was carried out in all three populations including N22/MTU1010 that was also genotyped through a whole-genome scan. Ten percent of the lines (5% with high GY and 5% with low GY under RS) were used for BSA. DNA of all the selected lines was extracted and quantified. DNA samples were bulked in equal quantity. Both bulks and two parents were screened with 140 polymorphic SSR markers for N22/Swarna and N22/IR64 and 125 polymorphic markers for N22/MTU1010 populations [26,42].

### Statistical analysis

Statistical analysis was done using SAS V9.1.3 [43]. Within a season, the performance of the i^th ^entry in the j^th ^block of the k^th ^replicate is modeled as-

(1) $$y_{ijk}=\mu +\alpha_{i}+r_{j}+b_{kj}+\varepsilon_{ijk}$$

where *μ *is the overall mean, *α _i _*is the effect of the i^th ^genotype, *r _j _*is the effect of the j^th ^replicate, *b _kj _*the effect of the k^th ^block within the j^th ^replicate and *ε _ijk _*the error. The genotypes were considered fixed while replicates and blocks within replicates were random. Line means and variance components were estimated using the MIXED procedure of SAS which uses the restricted maximum likelihood algorithm (REML) for estimating the variance components. REML [44] is a variant of the maximum likelihood (ML) estimation wherein the estimators are obtained not from maximizing the whole likelihood function but after removing the effect of the fixed effects. The variance components are less biased as they are estimated without being affected by the fixed effects and after taking into account the loss of degrees of freedom due to the fixed effects [45].

The conventional combined analysis of variance across seasons was first done with lines considered random and seasons considered fixed and replicates within sites and blocks within replicates within sites as random to estimate best linear unbiased estimates (BLUE) of the line means across seasons. For a given stress level, the performance of the i^th ^genotype in the k^th ^block of the j^th ^replicate of the l^th ^season is modeled as-

(2) $$y_{iljk}=\mu +\beta_{l}+r_{jl}+b_{kjl}+\alpha_{i}+\alpha \beta_{il}+\varepsilon_{ijk}$$

where *μ *is the overall mean, *β _l _*is the effect of the l^t h ^season, *r _jl _*is the effect of the j^th ^replicate within the l^th ^season, is *b _kjl _*the effect of the k^th ^block within the j^th ^replicate of the l^th ^season, *α _i _*is the effect of the i^th ^genotype, *αβ _il _*is the effect of interaction between the i^th ^genotype and the l^th ^season and *ε _ijk _*the error. To estimate the homogeneity of error variances, models assuming homogeneous and heterogeneous error variances were evaluated. Best fit model was used for combined mean analysis.

For estimating broad sense heritability, variance components were estimated considering all factors including genotypes as random. For each group, broad-sense heritability or repeatability (*H*) for each season was calculated as

$$H=\sigma_{g}^{2}∕\sigma_{p}^{2}\;\;\text{and}\;\;\sigma_{p}^{2}=\sigma_{g}^{2}+\left(\sigma_{e}^{2}∕r\right)$$

Where $\sigma_{p}^{2}$ is the phenotypic variance, $\sigma_{g}^{2}$ is the genotypic variance, $\sigma_{e}^{2}$ is the error variance and r is the number of replications in the season. Flowering delay was determined using entry means of two years. It was calculated as the difference between mean days to flowering under RS and NS.

To estimate the mean values of different marker classes, data were analyzed using a model in which marker classes were considered fixed and lines within marker classes as random using the REML algorithm of PROC MIXED of SAS.

To eliminate the confounding effect of DTF and PH on GY under RS, covariate analysis was carried using mean GY, DTF and PH data of two years under RS. The predicted mean GY after covariate adjustment was used for single-marker analysis to estimate the significance of differences between marker classes.

### Linkage map construction and QTL analysis

Genetic map distances between markers on all chromosomes in the N22/MTU1010 population were estimated using Mapdisto software v1.7 [46] with 362 genotypes and 120 markers. Five markers showing segregation distortion were omitted during map construction. In N22/Swarna and N22/IR64 populations, maps were constructed for markers used on chromosomes 1 and 3 and on chromosomes 1 and 2, respectively. A chi-square test (P < 0.01) was used to identify markers with distorted segregation. A LOD (logarithm of odds) value of 2.5 and map function Kosambi were used to estimate map distances.

Mixed model-based composite interval mapping in all three populations was carried out using QTL network v2.1 [47]. Marker intervals were determined and candidate intervals were selected. These selected intervals were used as a cofactor in a one-dimensional genome scan. An experiment-wise significance level for the determination of candidate intervals as well as putative QTL detection was set to a probability level of 0.01. A similar p-value was used for testing the significance of QTLs effects. For the estimation of threshold, 1000 permutation tests were performed. A window size of 10 cM and walk speed of 1 cM were used in the genome scan. Single-marker regression analysis to determine the phenotypic variance explained by each marker was carried out using QGene software [48]. QTL analysis was carried out with phenotypic data of both DS2009 and DS2010 separately for all three populations as well as with combined line means across both years. Identified QTLs were named as per the nomenclature procedure reported by McCouch [49].

## Results

### Phenotypic analysis of parents and populations

N22, the drought-tolerant donor, out-yielded three susceptible parents, Swarna, IR64, and MTU1010, under RS in both years. A reduction in mean GY under RS compared with mean GY under NS ranged between 57% and 60% in DS2009 and 68% and 82% in DS2010 in all three populations. Mean values of GY, DTF, PH, HI, and BIO in RS and NS experiments of DS2009 and DS2010 are presented in Table 1.

**Table 1:** Mean and range for yield and yield component traits in three populations and parents in DS2009 and DS2010

Population	Year/environment	DTF (days)			PH (cm)			BIO (kg/ha)			GY (kg/ha)			HI		
		
		Range	SED	Mean	Range	SED	Mean	Range	SED	Mean	Range	SED	Mean	Range	SED	Mean
N22/Swarna	2009RS	59-95	4	79	57-104	11.0	111.0	969-18973	3512	8854	178-5996	988	1714	0.02-0.39	0.07	0.18
	2009NS	67-99	4	77	57-163	11.0	119.0	3855-20953	2844	10700	1493-8118	1352	4313	0.18-0.60	0.06	0.38
	2010RS	69-108	5	86	41-98	8.1	75.0	11820-25664	1440	5517	122-1656	290	767	0.03-0.49	0.07	0.18
	2010NS	73-101	5	83	56-132	8.8	101.0	3700-25664	3166	9664	717-7564	902	3669	0.07-0.48	0.08	0.28

N22/IR64	2009RS	66-91	3	79	64-147	9.2	115.0	1629-15112	2540	7288	213-5113	665	1698	0.02-0.39	0.12	0.22
	2009NS	69-95	3	78	69-165	15.0	125.0	3625-24550	3820	12360	322-8055	1533	4200	0.11-0.48	0.05	0.33
	2010RS	73-99	4	88	41-107	9.1	71.2	1829-7217	1067	4152	65-955	186	423	0.01-0.32	0.06	0.09
	2010NS	74-91	4	83	59-132	16.0	98.0	4475-14221	2391	9084	1393-6924	1181	3861	0.09-0.45	0.06	0.26

N22/MTU1010	2009RS	69-91	3	78	61-149	12.0	113.0	3290-25015	3058	8938	40-6135	831	2304	0.06-0.40	0.07	0.23
	2009NS	71-93	5	77	76-173	16.0	127.0	2452-28583	4197	12870	652-10042	1693	4774	0.14-0.52	0.06	0.35
	2010RS	72-95	4	85	43-106	9.4	76.0	1872-8300	1329	4641	34-1302	211	653	0.01-0.29	0.05	0.06
	2010NS	74-89	3	82	61-133	21.0	104.0	2692-19384	2384	9051	1245-8308	1162	4091	0.10-0.51	0.07	0.29

N22	RS			72			115.0			5818			1150			0.21
	NS			78			123.0			9087			3221			0.41

Swarna	RS			a			59.0			6125			128			0.03
	NS			107			98.0			11126			4144			0.31

IR64	RS			91			73.0			4175			336			0.03
	NS			89			105.0			10661			4651			0.46

MTU1010	RS			84			95.0			4181			629			0.11
	NS			83			110.0			11337			4925			0.49

• Range for the parents was given based on the data of two years,^a^Swarna did not flowered up to 50% under RS.

• SED: Standard error of difference at p < 0.05

Correlations of GY with yield-attributing traits were calculated (Table 2). GY was positively correlated with PH, HI, and BIO in both RS and NS environments. DTF was negatively correlated with GY under RS in both dry seasons in all three populations, except for N22/Swarna in DS2010, and was positively correlated with GY under NS in both dry seasons in all populations except for N22/MTU1010 in DS2009.

**Table 2:** Correlation of GY with yield component traits and heritability of GY and related traits under RS and NS in DS2009 and DS2010

Correlation						Heritability				
**Population**	**Year**	**DTF**	**PH**	**BIO**	**HI**	**DTF**	**PH**	**BIO**	**GY**	**HI**

N22/Swarna	2009RS	-0.42**	0.51**	0.63**	0.64**	0.66	0.86	0.53	0.54	0.56
	2009NS	0.23**	0.33**	0.54**	0.26**	0.81	0.85	0.54	0.55	0.54
	2010RS	-0.05	0.35**	0.25**	0.48**	0.83	0.78	0.38	0.50	0.53
	2010NS	0.18*	0.35**	0.46**	0.25**	0.82	0.84	0.58	0.66	0.39

N22/IR64	2009RS	-0.36**	0.43**	0.68**	0.18**	0.74	0.93	0.45	0.67	0.73
	2009NS	0.04	0.54**	0.67**	0.41**	0.91	0.83	0.58	0.63	0.62
	2010RS	-0.40**	0.25**	0.26**	0.56**	0.76	0.94	0.23	0.56	0.57
	2010NS	0.23**	0.50**	0.53**	0.44**	0.77	0.84	0.48	0.67	0.58

N22/MTU1010	2009RS	-0.52**	0.35**	0.50**	0.54**	0.80	0.92	0.37	0.56	0.67
	2009NS	-0.11	0.25**	0.58**	0.35**	0.60	0.74	0.46	0.61	0.61
	2010RS	-0.42**	0.29**	0.25**	0.49**	0.76	0.75	0.34	0.62	0.75
	2010NS	0.35**	0.19*	0.62**	0.50**	0.76.	0.77	0.40	0.76	0.45

*Significance at 5%; **Significance at 1%.

Broad-sense heritability (*H*) of traits measured in an individual year in all three populations ranged from 0.23 to 0.94 (Table 2). Heritability of GY under RS ranged from 0.50 to 0.67 in DS2009 and DS2010. Under NS, it ranged from 0.55 to 0.76. The heritability of other yield-related traits is presented in Table 2.

### Genotypic analysis of mapping populations

**Figure 1:**
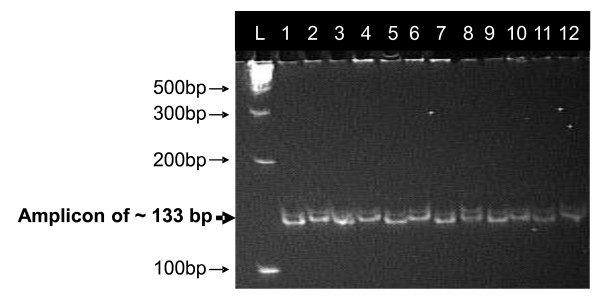
**Bulk segregant analysis in three populations with RM315**. L: 100 bp ladder; 1. N22; 2. IR64; 3. Bulk of high-yielding lines in N22/IR64 population; 4. Bulk of low-yielding lines in N22/IR64 population; 5. N22; 6. MTU1010; 7. Bulk of high-yielding lines of N22/MTU1010 population; 8. Bulk of low-yielding lines of N22/MTU1010 population; 9. N22; 10. Swarna; 11. Bulk of high-yielding lines of N22/Swarna population; 12. Bulk of low-yielding lines of N22/Swarna population. Arrows indicate difference between high-yielding and low-yielding bulks.

**Figure 2:**
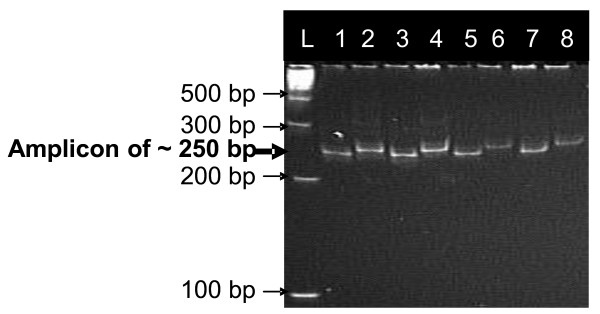
**Bulk segregant analysis in N22/IR64 and N22/MTU1010 populations with RM431**. L; 100 bp ladder; 1. N22; 2. IR64; 3. Bulk of high-yielding lines in N22/IR64 population; 4. Bulk of low-yielding lines in N22/IR64 population; 5. N22; 6. MTU1010; 7. Bulk of high-yielding lines of N22/MTU1010 population; 8. Bulk of low-yielding lines of N22/MTU1010 population. Arrows indicate difference between high-yielding and low-yielding bulks.

**Figure 3:**
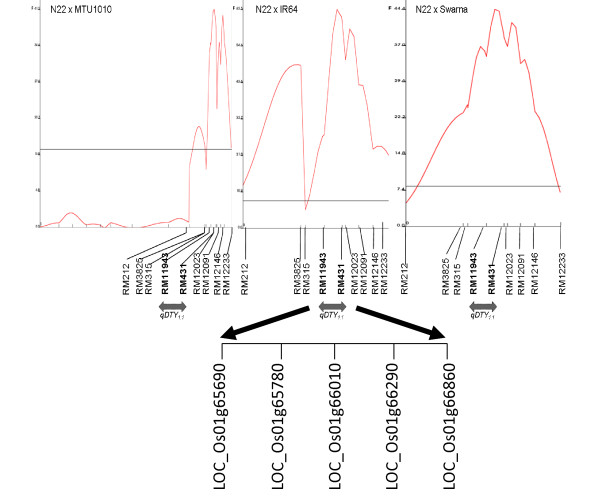
***F*-statistic curve representing QTL *qDTY_1.1 _*in three populations for yield under drought stress (combined analysis)**. Flanking and peak markers mentioned on X-axis and F-values on Y-axis (F-values also given in Table 3). Peak of qDTY_1.1 _was between RM11943 and RM431 in combined analysis. Figure also presents differentially expressed genes between N22 and IR64 in *qDTY*_*1.1 *_region.

Polymorphism of N22 with Swarna, IR64, and MTU1010 was 43.5%, 42.5%, and 40.1%, respectively. The N22/MTU1010 population was genotyped with 125 polymorphic SSR markers. BSA was carried out in all three populations with identified polymorphic markers. In BSA, RM315 showed a different banding pattern between parents and two bulks in all three populations while RM212, RM431, and RM12233 showed differences in N22/IR64 and N22/MTU1010 populations. The banding pattern of high-yield bulks corresponded to N22 and that of low-yield bulks corresponded to Swarna/IR64/MTU1010 for RM315 (Figure 1). The banding pattern of N22 and IR64/MTU1010 corresponded well with high-yield and low-yield bulks for RM212, RM431, and RM12233 (Figure 2). The markers showing differences in banding pattern and additional markers (RM3825, RM11943, RM12023, RM12091, and RM12146) were run on all the lines in three populations (Figure 3). A linkage map of the N22/MTU1010 population with 120 polymorphic SSR markers covered a map distance of 1521 centiMorgans (cM) (Additional file 1). Similarly, markers used for N22/IR64 (chromosomes 1 and 2) and N22/Swarna (chromosomes 1 and 3) populations covered map distances of 61.9 and 49.45 cM (Additional files 2 and 3). QTLs for GY, DTF, PH, HI, and BIO were identified. Results of QTL analysis are presented in Tables 3 and 4.

**Table 3:** QTLs for yield and yield component traits in three populations under reproductive-stage drought stress in DS2009 and DS2010

Population	Trait	QTL	Chromosome	Peak interval	F-value			Additive effect (%)			R^2^		
					
					2009	2010	Combined	2009	2010	Combined	2009	2010	Combined
N22/Swarna	DTF	*qDTF_1.1_*	1	RM11943-RM431	21.99	10.35	17.85	-2.7	-2.2	-2.3	5.2	2.0	3.5
		*qDTF_3.2_*	3	RM60-RM22	79.62	135.54	134.86	-4.3	-8.2	-6.3	23.4	33.3	34.9
	PH	*qDTH_1.1_*	1	RM11943-RM12091	124.03	60.30	120.30	12.9	9.0	11.8	38.6	21.7	32.6
	BIO	*qDTB_1.1_*	1	RM315-RM431	67.45	8.12	65.47	30.8	6.4	22.6	20.1	4.4	20.1
		*qDTB_3.2_*	3	RM60-RM22		24.61			-10.2			8.6	
	GY	*qDTY_1.1_*	1	RM11943-RM12091	42.89	11.03	40.83	35.5	9.4	29.3			13.4
		*qDTY_3.2_*	3	RM60-RM22	17.14		15.08	13.6		12.9	3.2		3.2
	HI	*qDTHI_1.1_*	1	RM11943-RM431	20.05			9.5			6.2		
		*qDTHI_3.2_*	3	RM60-RM22	73.62	13.52	61.97	19.9	11.1	20.1	12.5	4.8	18.5

N22/IR64	DTF	*qDTF_1.1_*	1	RM11943-RM431	20.43	17.71	22.56	-1.2	-2.1	-1.7	7.1	6.2	7.6
	PH	*qDTH_1.1_*	1	RM11943-RM431	280.28	132.25	223.48	17.6	8.7	10.4	58.0	36.9	51.8
	BIO	*qDTB_1.1_*	1	RM11943-RM431	91.73		85.00	24.2		16.1	24.3		22.6
	GY	*qDTY_1.1_*	1	RM11943-RM12091	69.16	11.17	57.61	30.0	10.1	24.3			16.9
		*qDTY_2.3_*	2	RM263-RM530		10.88			9.6			4.6	
	HI	*qDTHI_2.3_*	2	RM13600-RM263		17.69	9.21		16.8	8.3		4.9	3.7

N22/MTU1010	DTF	*qDTF_1.1_*	1	RM11943-RM431	10.14	27.32	23.17	1.0	-2.0	-1.4	1.6	6.9	5.5
		*qDTF_3.3_*	3	RMRM517-RM232	15.33			-1.1			2.6		
		*qDTF_10.1_*	10	RM271-RM304	55.21	45.50	65.29	2.4	2.5	2.5	9.8	8.9	11.8
	PH	*qDTH_1.1_*	1	RM11943-RM431	284.32	177.08	307.80	16.1	14.2	15.3	5.3	3.7	53.5
	BIO	*qDTB_1.1_*	1	RM11943-RM431	127.94	28.74	149.47	21.6	9.6	17.6	22.3	7.6	30.3
	GY	*qDTY_1.1_*	1	RM11943-RM12091	36.77	18.47	40.04	18.1	9.6	16.1			12.6
		*qDTY_10.1_*	10	RM216-RM304	19.94	15.68	24.20	-13.2	-12.6	-12.2	3.7	3.0	5.0

**Table 4:** QTLs for yield and yield component traits in three populations under non-stress in DS2009 and DS2010

Population	Trait	QTL	Chromosome	Peak interval	F- value			Additive effect (%)			R^2^		
					
					2009	2010	Combined	2009	2010	Combined	2009	2010	Combined
	DTF	*qDTF_3.2_*	3	RM60-RM22	113.21	125.51	155.93	-5.6	5.3	-5.4	33.3	34.5	38.0
	PH	*qDTH_1.1_*	1	RM11943-RM12091	70.30	38.56	82.31	12.2	7.5	10.4	24.8	15.2	27.2
		*qDTH_3.2_*	3	RM60-RM231	10.91	12.55	17.20	-3.5	-3.9	-4.2	3.6	4.2	5.1
N22/Swarna	BIO	*qDTB_1.1_*	1	RM11943-RM12091	20.13		14.21	9.1		7.8	8.6		5.6
		*qDTB_3.2_*	3	RM60-RM22	39.27	58.69	67.63	-12.0	-20.5	-16.1	12.1	14.9	18.5
	GY	*qDTY_1.1_*	1	RM11943-RM12091	18.60	17.06	21.07	13.6	12.1	13.0			8.8
	HI	*qDTHI_3.2_*	3	RM60-RM489	8.16	36.36	19.83	3.8	10.7	6.1	2.6	11.8	5.1

	PH	*qDTH_1.1_*	1	RM11943-RM12091	236.02	117.17	121.30	12.3	7.9	5.8	51.8	35.5	51.3
N22/IR64	BIO	*qDTB_1.1_*	1	RM11943-RM12091	90.93	8.94	77.30	20.2	4.3	13.1	27.3	5.3	25.0
	GY	*qDTY1.1*	1	RM11943-RM12091	60.92	15.64	59.17	22.1	7.1	15.1			18.9
	HI	*qDTHI_1.1_*	1	RM11943-RM12091		11.84			-7.8			3.6	

	DTF	*qDTF_10.1_*	10	RM271-RM304	42.58	57.79	62.84	2.6	2.0	2.3	13.1	5.4	11.1
	PH	*qDTH_1.1_*	1	RM11943-RM12091	236.53	73.98	270.27	13.8	12.0	12.47	45.7	29.7	50.6
N22/MTU1010	BIO	*qDTB_1.1_*	1	RM11943-RM12091	62.51	14.99	28.70	16.9	7.7	7.9	15.0	5.1	0.8
	GY	*qDTY1.1*	1	RM11943-RM12091	19.07			12.1					
	HI	*qDTHI_1.1_*	1	RM11943-RM12091		13.76			-3.4			5.5	

### QTLs for yield and yield related traits

A total of four QTLs were identified for GY under RS in all three populations. QTL *qDTY_1.1 _*with a large and consistent effect in all three populations was identified on chromosome 1. Three other QTLs, *qDTY_2.3_*, *qDTY_3.2 _*and *qDTY_10.1_*, specific to individual populations/environments were identified. The allele increasing GY under RS was contributed by N22 in *qDTY_1.1_*, *qDTY_2.3_*, and *qDTY_3.2 _*and by MTU1010 in *qDTY_10.1_*. Four QTLs for DTF were identified in three populations--*qDTF_1.1_*, *qDTF_3.2_*, *qDTF_3.3_*, and *qDTF_10.1_*. Under RS, *qDTF_1.1_*, *qDTF_3.2_*, *qDTF_3.3_*, and *qDTF_10.1 _*showed significant effects. *qDTF_1.1 _*did not show effect in NS in any of the three populations. Two QTLs (*qDTB_1.1 _*and *qDTB_3.2_*) were identified for biomass increase under RS and NS (Tables 3 and 4). For PH *qDTH_1.1 _*and *qDTH_3.2 _*were significant. *qDTHI_3.2 _*showed significant effect on harvest index in N22/Swarna population.

QTLs *qDTB_1.1_*, *qDTH_1.1_*, and *qDTF_1.1 _*co-localized on chromosome 1 with *qDTY_1.1 _*region in all three populations. In N22/IR64 population *qDTY_2.3 _*and *qDTHI_2.3 _*co-localized on chromosome 2. Similarly, *qDTY_3.2_*, *qDTB_3.2_*, *qDTH_3.2_*, *qDTHI_3.2_*, and *qDTF_3.2 _*co-localized on chromosome 3 in N22/Swarna.

In combined QTL analysis over two years as well as in individual year analysis, *qDTY_1.1 _*located on the distal end of chromosome 1 spanned from RM212 to RM12233 in all three populations (Figure 3). The peak in all three populations was flanked by markers RM11943 and RM431. Under RS, *qDTY_1.1 _*explained phenotypic variance (R^2^) of 13.4%, 16.9%, and 12.6% in N22/Swarna, N22/IR64, and N22/MTU1010 populations, respectively. The additive effect (additive effect as % of trial mean) of this QTL for GY under RS was 29.3%, 24.3%, and 16.1% of the respective means in N22/Swarna, N22/IR64, and N22/MTU1010 populations (Table 3).

In NS conditions, *qDTY_1.1 _*showed a consistent effect over two years in N22/Swarna and N22/IR64 populations as well as in the N22/MTU1010 population in DS2009 (Table 4). Phenotypic variance explained by this QTL was 8.8%, with an additive effect of 13.0% of the population mean in N22/Swarna, whereas phenotypic variance was 18.9%, with an additive effect of 15.1% of the population mean in N22/IR64 (Table 4). This is the first reported major QTL with a consistent effect on GY under both RS and NS situations across elite multiple genetic backgrounds.

*qDTF_1.1 _*explained phenotypic variance of 3.5%, 7.6%, and 5.5% in N22/Swarna, N22/IR64, and N22/MTU1010 populations, respectively (Table 3). The phenotypic variance explained by *qDTB_1.1 _*under RS conditions was 20.1%, 22.6%, and 30.3% in N22/Swarna, N22/IR64, and N22/MTU1010 populations, respectively. Under NS, *qDTB_1.1 _*explained phenotypic variance of 5.6%, 25.0%, and 0.8% in N22/Swarna, N22/IR64, and N22/MTU1010 populations, respectively. The additive effect contributed by *qDTB_1.1 _*in N22/Swarna, N22/IR64, and N22/MTU1010 populations was 22.6%, 16.1%, and 17.6% of the population mean under RS. The additive effect under NS was 7.8%, 13.1%, and 7.9% in N22/Swarna, N22/IR64, and N22/MTU1010 populations, respectively. *qDTH_1.1 _*had a significant effect on PH under RS and NS conditions in all three populations over both years. Under RS, *qDTH_1.1 _*explained phenotypic variance of 32.6%, 51.8%, and 53.5% in N22/Swarna, N22/IR64, and N22/MTU1010 populations, respectively (Table 3). Under NS, *qDTH_1.1 _*explained phenotypic variance of 27.2%, 51.3%, and 50.6% in N22/Swarna, N22/IR64, and N22/MTU1010 populations, respectively (Table 4).

In addition to QTLs on chromosome 1, the study identified some other QTLs for GY and yield related traits on chromosome 2, 3 and 10 (Tables 3 and 4). *qDTY*_*2.3 *_was significant in DS2010 only under RS in N22/IR64 population. Similarly, *qDTY*_*3.2 *_was significant in DS2009 under RS in N22/Swarna population. Collocating QTL *qDTB_3.2 _*showed a significant effect in DS2010 under RS. At same locus, *qDTH_3.2_*, showed a consistent effect on PH over two years under NS. *qDTHI_3.2 _*significantly increased harvest index under RS as well as in NS conditions across the years. The phenotypic variance explained by this QTL under RS and NS was 18.5% and 5.1%, respectively (Tables 3 and 4). *qDTF*_*3.2 *_showed a significant effect in NS condition in both years. A locus on chromosome 10, *qDTY_10.1 _*was identified in N22/MTU1010 population for DTF and GY under RS. *qDTF_10.1 _*showed significant effect over years under RS and NS. *qDTY_10.1 _*was significant under RS and positive allele was contributed from comparatively less susceptible parent MTU1010 (Table 3 and 4).

### Effect of DTF and PH on the performance of *qDTY_1.1_*

To eliminate the confounding effect of DTF on GY under RS, covariate analysis was carried out taking days to flowering as a co-factor. After eliminating the confounding effect of DTF, mean GY was predicted using the mean GY of each year under RS in all three populations. Single-marker analysis was carried out using predicted GY with RM11943 and RM431 (Table 5). Both markers were highly significant for GY under RS even after covariate adjustment. In addition, all three populations were divided into two subsets based on their range of mean DTF (one with 70-79 days and the other with 80-90 days). Each subset was subjected to composite interval mapping as well as single-marker analysis with the *qDTY_1.1 _*flanking markers RM11943 and RM431. Within these subsets, mean GY of the N22 homozygote was significantly higher than that of the Swarna homozygotes (data not presented). These results indicate that an increase in GY by *qDTY_1.1 _*is not due to early flowering under RS. Moreover, the delay in flowering of lines without *qDTY_1.1 _*(Swarna/IR64/MTU1010 homozygote for the marker closest to the peak) was more than in those with *qDTY_1.1 _*(N22 homozygote for the marker closest to the peak). The mean flowering delay of lines without *qDTY_1.1 _*was +2.6, +1.4, and +1.3 days compared with +0.5, -0.1, and -0.3 day in lines with *qDTY_1.1 _*in N22/Swarna, N22/IR64, and N22/MTU1010 populations, respectively.

**Table 5:** Single-marker analysis for the effect of *qDTY_1.1 _*on grain yield after covariance adjustment for effect of days to 50% flowering under drought stress in three populations

Population	Marker	Mean grain yield of N22 homozygotes (kg/ha)	Mean grain yield of IR64, Swarna, MTU1010 homozygotes (kg/ha)	p-value
N22/IR64	RM431	1273	761	< 0.001
	RM11943	1239	878	< 0.001
N22/Swarna	RM431	1517	926	< 0.01
	RM11943	1484	927	< 0.01
N22/MTU1010	RM431	1543	1149	< 0.01
	RM11943	1531	1199	< 0.01

Homozygotes and heterozygotes determined based on the genotype of RM11943 and RM431.

P < 0.01, highly significant

Covariance analysis was also carried for PH using two peak markers RM11943 and RM431. Even after co-variance adjustment, *qDTY_1.1 _*showed significant effect on GY under RS in two out of three populations (Table 6).

**Table 6:** Single-marker interval (RM11943-RM431) analysis for the effect of *qDTY_1.1 _*on GY after covariance adjustment for plant height under RS in three mapping populations based on the mean grain yield over two years

Population	Mean grain yield of N22 homozygotes*	Mean grain yield of IR64/, Swarna/, MTU1010 homozygotes	p-value
N22/Swarna	1448	1267	< 0.01
N22/IR64	1330	1073	< 0.01
N22/MTU1010	1470	1381	NS

*Determined based on the genotypes of RM11943 and RM431.

## Discussion

Breeding for drought tolerance is a high-priority area of rice research for sustainable production as the severity and frequency of drought occurrences are expected to increase because of the ongoing climatic change process [3,4]. There is an urgent need to breed drought-tolerant rice varieties with high yield potential. Over the last few years, two to three varieties in each country have become popular among farmers and are grown on millions of hectares because of their high yield potential and preferred grain quality. But, these varieties are highly susceptible to RS. The adaptability of such farmer-preferred varieties could be increased by incorporating drought tolerance. The identification and introgression of QTLs for GY under RS from landraces and wild progenitor species in popular rice varieties is a fast-track approach in breeding drought-tolerant rice varieties.

Our study aimed at identifying QTLs with a major and consistent effect on GY under RS from a drought-tolerant donor, N22, a selection from landrace Rajbhog. This donor is well known for its drought tolerance and for maintaining high spikelet fertility even under drought and heat stress [50,51].

The mean reduction in GY under RS ranged from 57.3% to 60.4% in DS2009 and from 79% to 89% in DS2010 in three populations, indicating the severity of RS in two-year screens. RS was more severe in DS2010 than in DS2009. In DS2009, 47.3 mm of rainfall were spread over 13 rainy days, whereas, in DS2010, 26 mm of rainfall were spread over just 7 rainy days, received from stress initiation to maturity. In two years of evaluation, N22, the tolerant parent, always out-yielded Swarna, IR64, and MTU1010 under RS, confirming its tolerance of drought. Among the four parents, Swarna was the most susceptible. It even failed to flower under RS in both years. Earlier, Swarna was reported to be severely affected by RS [26]. IR64, the popular rice variety of Asia, where 90% of the world's rice is grown, exhibited a 92% and 89% reduction in GY compared with GY in NS in DS2009 and DS2010. IR64 has also been reported to be highly susceptible to drought [33]. MTU1010, the fourth parent under study, exhibited an 87% and 88% reduction in GY compared with NS yields in DS2009 and DS2010.

The traits observed in our study showed moderate to high broad-sense heritability in all three populations and in both years. Further, the heritability for GY under RS was similar to that under NS in both years, indicating that selection for GY under RS has practical applicability. Heritability was highest for DTF and PH. GY under RS was reported to be a highly heritable trait in previous studies also [8,19,25]. Selection for GY under RS is now a well-recommended selection criterion for breeding drought-tolerant rice varieties [8].

For GY under RS, four QTLs were identified. It is interesting to note that we found a QTL on chromosome 1 (*qDTY_1.1_*) with a major and consistent effect against all three improved genetic backgrounds and in both dry seasons. *qDTY_1.1 _*showed a large effect on yield in all three populations in DS2009, with the effect being highest in N22/Swarna, the parent with the highest drought susceptibility. Further, the effect was lowest in the N22/MTU1010 population, the parent with the least drought susceptibility among the three. In DS2010, under more severe RS, the effect was similar against all three genetic backgrounds. Combined over two years, the effect was highest in the Swarna background, followed by IR64 and MTU1010 backgrounds.

QTLs from tolerant donors identified to show high effect against non-elite drought susceptible genetic backgrounds are less likely to show similar high effect against high yielding, elite genetic backgrounds [24]. Further, QTLs from particular genetic backgrounds usually show smaller effects or disappear altogether in different genetic backgrounds [24]. In the case of drought, QTL × genetic background interaction has been reported to be a major factor limiting the use of QTLs for MAB in rice [27,28,30]. *qDTY_1.1 _*has been identified in the background of highly popular elite genetic backgrounds, shows consistent effects against different genetic backgrounds, and is suitable for use in MAB to improve popular high-yielding varieties.

In combined analysis over two years, the phenotypic variance explained by this QTL was maximum in the N22/IR64 population, followed by N22/Swarna and N22/MTU1010 (Table 3). The additive effect of the QTL as a percent of trial means under RS was highest in N22/Swarna (29.3%) and lowest in N22/MTU1010 (16.1%). The phenotypic variance and additive effect explained by *qDTY_1.1 _*were lowest in the N22/MTU1010 population in both years. These findings clearly suggest that the use of diverse and contrasting parents for population development could lead to the identification of large-effect QTLs. With two earlier identified major QTLs for GY under RS (*qDTY_12.1 _*and *qDTY_3.1_*) reported recently, contrasting parents were also used [19,26].

It is noteworthy to mention here that *qDTY_1.1 _*had a significant effect on GY under both RS and NS conditions in all three backgrounds in both seasons, except in the N22/MTU1010 population in DS2010. The additive effect of *qDTY_1.1 _*in NS was smaller than that under RS but even then it was between 7.1% and 22.1% of the trial mean yield in all populations. A QTL for GY under RS (*qDTY_3.1_*) reported earlier by Venuprasad et al. [26] showed a negative effect on GY under NS conditions. *qDTY_1.1 _*is the first reported QTL with a major and consistent positive effect on GY under both RS and NS situations in multiple genetic backgrounds and it is a QTL fit to simultaneously increase yield under both situations.

Three populations were screened at IRRI in DS2009 and DS2010. The dry season screening at IRRI is comparable to wet season screening in India as reported by earlier workers [22]. However screening of these populations at multiple locations would add value to the QTL validation across different target environments.

*qDTY_1.1 _*co-located with DTF under RS. To remove the confounding effect of DTF on GY, covariance adjustments were made [26]. Also, after covariance adjustment of DTF, *qDTY_1.1 _*showed a significant effect on GY under RS in all three populations (Table 5). Further, each of the three populations was classified into two subsets based on their mean DTF, one with a DTF range of 70-79 days and the other with 80-90 days. The effect of RM11943 and RM431 on GY under RS in these two subsets of populations was significant in all populations (data not presented). Further, this QTL was non-significant for DTF in all three populations under NS environments of both years. These facts rules out the possibility of this QTL being responsible for drought escape at RS.

*qDTY_1.1 _*showed a significant effect for PH under RS and NS in all three populations in both seasons. This QTL showed significant effect on GY under RS even after covariance adjustment of PH in N22/Swarna and N22/IR64 populations (Table 6). *qDTY_1.1 _*also showed a significant effect on BIO increase under RS and NS situations except in N22/IR64 in DS2010 under RS and in N22/Swarna in DS2010 under NS. Our results clearly indicated that the increase in GY under both RS and NS by *qDTY_1.1 _*was mainly due to an overall increase in BIO. Bernier et al. [19] and Kumar et al. [25] also reported the contribution of an increase in BIO to an increase in GY under RS. QTLs for various drought-tolerance traits, including root traits [52], PH [19], root dry weight [53], grains per panicle [18], relative water content under drought [54], biomass, basal root thickness, and osmotic adjustment [27,55], have also been reported in the same region. QTLs for GY under RS in this region have also been reported by Kumar et al. [25] and the allele increasing GY under RS was contributed by a comparatively susceptible parent; however, the positive allele in our study was contributed by the tolerant parent, N22. Also, the QTL identified in our study showed effect under NS unlike other QTLs identified at same locus by earlier workers [25]. QTLs for flag-leaf net photosynthetic rate, stomata frequency, heading date under RS, and PH under RS were also reported by earlier workers in this region [56,57].

Our study also identified additional QTLs for GY under RS that showed an effect against a single genetic background. *qDTY_2.3 _*in N22/IR64, *qDTY_3.2 _*in N22/Swarna, and *qDTY_10.1 _*in N22/MTU1010 populations were identified. *qDTY_3.2 _*had a significant effect on GY under RS in DS2009 only, whereas *qDTY_10.1 _*showed an effect in both seasons. These two QTLs also co-located with QTLs for DTF under RS as well as NS conditions in both seasons.

*qDTY_2.3 _*on chromosome 2 in the N22/IR64 population significantly increased GY under severe RS of DS2010 only and the allele increasing GY was contributed by N22. This QTL also increased HI under RS in DS2010. This region has been reported to be associated with various drought-tolerance traits such as panicle number [19], leaf rolling [52], and osmotic adjustment [53].

A reduction in DTF by *qDTY_3.2_*, contributed by N22, an early-duration variety, was observed in our study. This region was significant for PH under NS in both seasons and under RS in DS2009. *HD9 *is already reported within this region for heading date in rice [58]. Earlier reports also suggest that this region is associated with DTF, BIO under RS, and PH under NS [1,19,56].

Interestingly, the positive allele for GY under RS in *qDTY_10.1 _*is contributed by MTU1010, which is a drought-susceptible parent in comparison with N22. Since this QTL is significant for DTF in both RS and NS situations, the increase in GY due to the QTL may be attributed to drought escape. To confirm this, covariance analysis was carried out to remove any confounding effect of DTF on GY, taking DTF as a co-factor. After the covariate adjustment, this QTL was not significant for GY under RS, confirming that the increase in GY under RS is due to a reduction in DTF.

N22 is categorized in the *aus *group of rice varieties [59] and it contributes an allele that increases GY under RS in *qDTY_1.1_*. It is worth mentioning that QTLs *qDTY_1.1_*, *qDTY_2.3_*, and *qDTY_3.2 _*in our study coincide with the QTLs for drought-related secondary traits in Vandana/Way Rarem population [19]. Vandana is an eastern Indian variety developed from C22/Kalakeri. Kalakeri also belongs to the *aus *group. These drought-tolerant regions likely accumulated in *aus *cultivars during evolution and are coming from *aus *backgrounds. Drought-tolerant cultivars such as N22 and Dular have been categorized in the *aus *cluster by genome-wide SNP-based diversity analysis [59].

Meta analysis of GY QTLs under RS has also emphasized the importance of this region for GY under drought in rice [60]. Within *qDTY_1.1 _*region (Figure 3), 4, 5-DOPA dioxygenase extradiol (LOC_Os01g65690), glycosyl transferase (LOC_Os01g65780), amino acid transporters (LOC_Os01g66010), MADS-box family gene (LOC_Os01g66290) and serine/threonine protein kinase (LOC_Os01g66860) have been recently reported as differentially expressed genes between N22 and IR64 [61]. These genes could be potential candidates for further genomics studies for drought tolerance. The consistent effect of *qDTY_1.1 _*in different elite genetic backgrounds makes it a suitable candidate for functional genomics and expression analysis studies to identify genes for GY under RS.

## Conclusions

QTL *qDTY_1.1 _*has been identified as a major QTL for GY under RS with a positive effect under NS conditions as well. The effect of this QTL on GY under RS is consistent in the backgrounds of three rice mega-varieties of rainfed lowland ecosystems of South and Southeast Asia, where 90% of the world's rice is grown. *qDTY_1.1 _*could be used effectively for improving the GY of lowland rice cultivars Swarna, IR64, and MTU1010 under both RS and NS situations. The consistent effect of *qDTY_1.1 _*across different genetic backgrounds opens up an opportunity for further functional genomics studies to identify genes for GY under RS and to develop gene-based markers. Further, *qDTY_1.1 _*can be pyramided with other reported QTLs for GY under RS in Swarna, IR64, and MTU1010 backgrounds to obtain an appreciable yield enhancement of at least 1.0 t ha^-1 ^under severe RS.

## List of Abbreviations

QTLs: Quantitative trait loci; RS: Reproductive-stage drought stress; NS: Non-stress; DS: Dry season; IRRI: International Rice Research Institute; MAB: Marker-assisted breeding; NPK: Nitrogen, phosphorus, and potassium; DAT: Days after transplanting; DTF: Days to 50% flowering; PH: Plant height; BIO: Biomass; GY: Grain yield; HI: Harvest index; DNA: Deoxyribonucleic acid; CTAB: Cetyl trimethyl ammonium bromide; PCR: Polymerase chain reaction; PAGE: Polyacrylamide gel electrophoresis; SSR: Simple sequence repeats; BSA: Bulked segregant analysis; LOD: logarithm of odds; R^2^: Phenotypic variance; SNP: Single nucleotide polymorphism.

## Authors' contributions

AK formulated the concept of QTL identification in three different populations simultaneously. PV developed three mapping populations with the help of MTSC. Phenotyping was done by PV and MTSC. PV was also in charge of genotyping. Statistical and QTL analysis were done by PV with the help of BPMS and SD. PV and BPMS drafted the manuscript and the other authors contributed to revisions for the final draft. All authors read and approved the final manuscript.

## Supplementary Material

Additional file 1 **Genetic linkage map of N22/MTU1010 population**. Click here for file

Additional file 2 **Genetic map distances among markers on chromosomes 1 and 2 in N22/IR64 population**. Click here for file

Additional file 3 **Genetic map distances among markers on chromosomes 1 and 3 in N22/Swarna population**. Click here for file
