# Supplementary figures and images for: qDTY1.1, a major QTL for rice grain yield under reproductive-stage drought stress with a consistent effect in multiple elite genetic backgrounds
Source: BMC Genet. 2011 Oct 18;12:89. doi: 10.1186/1471-2156-12-89 (PMC3234187; doi:10.1186/1471-2156-12-89)

## Slide 1
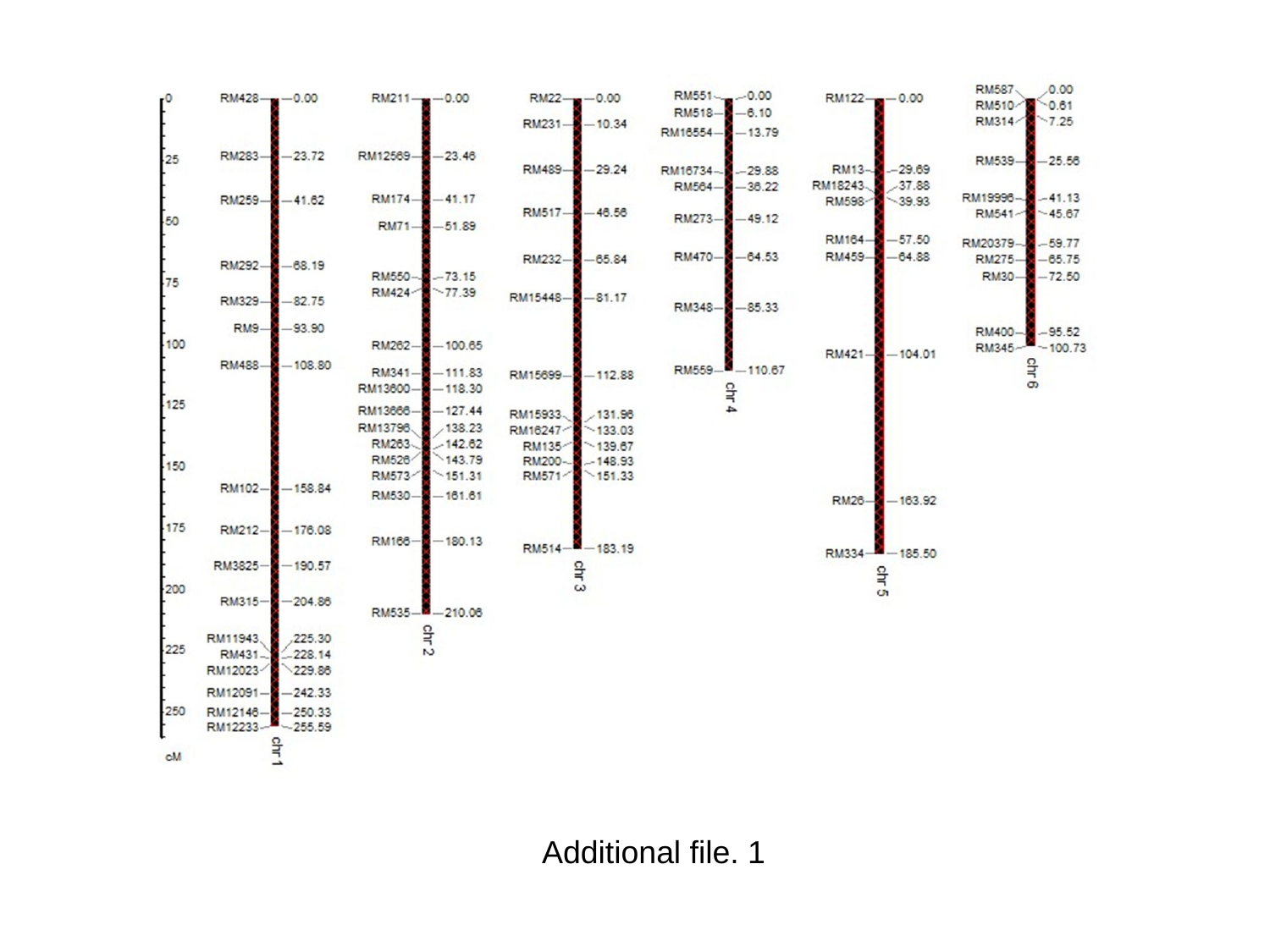

Additional file. 1

## Slide 2
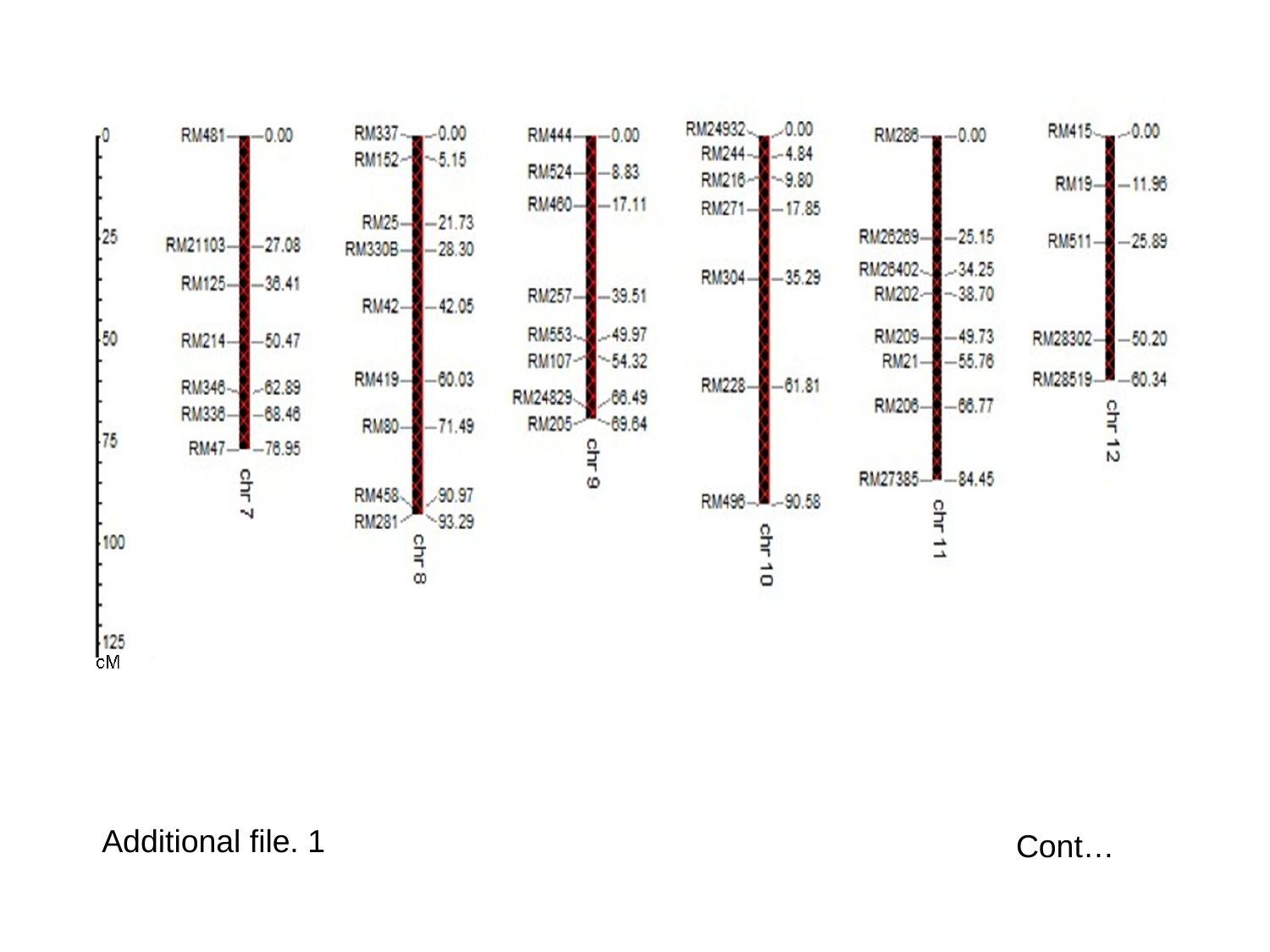

Additional file. 1
Cont…

Supplement: Additional file 1 — Genetic linkage map of N22/MTU1010 population. [file 1471-2156-12-89-S1.PPT]

## Slide 1
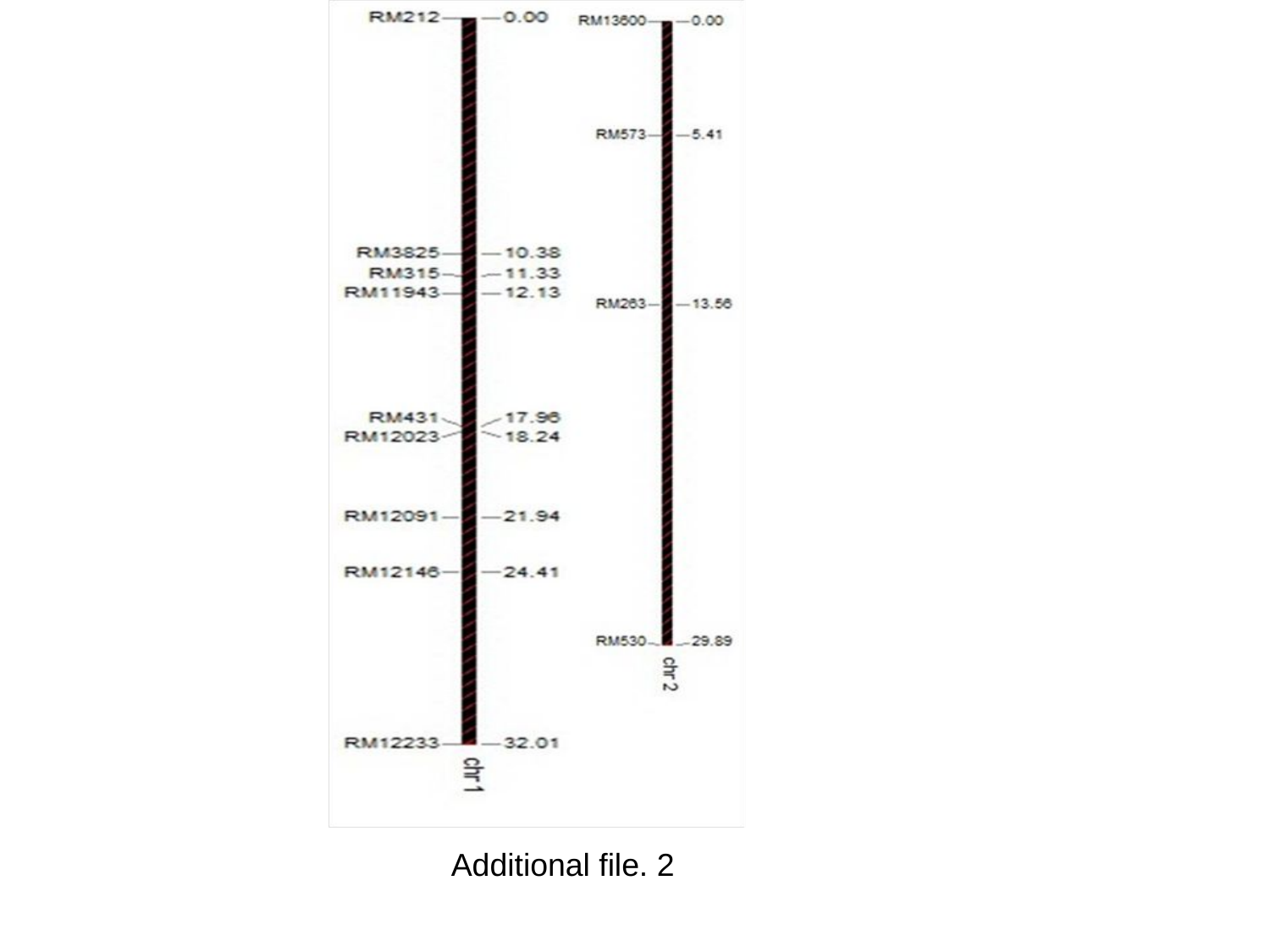

Additional file. 2

Supplement: Additional file 2 — Genetic map distances among markers on chromosomes 1 and 2 in N22/IR64 population. [file 1471-2156-12-89-S2.PPT]

## Slide 1
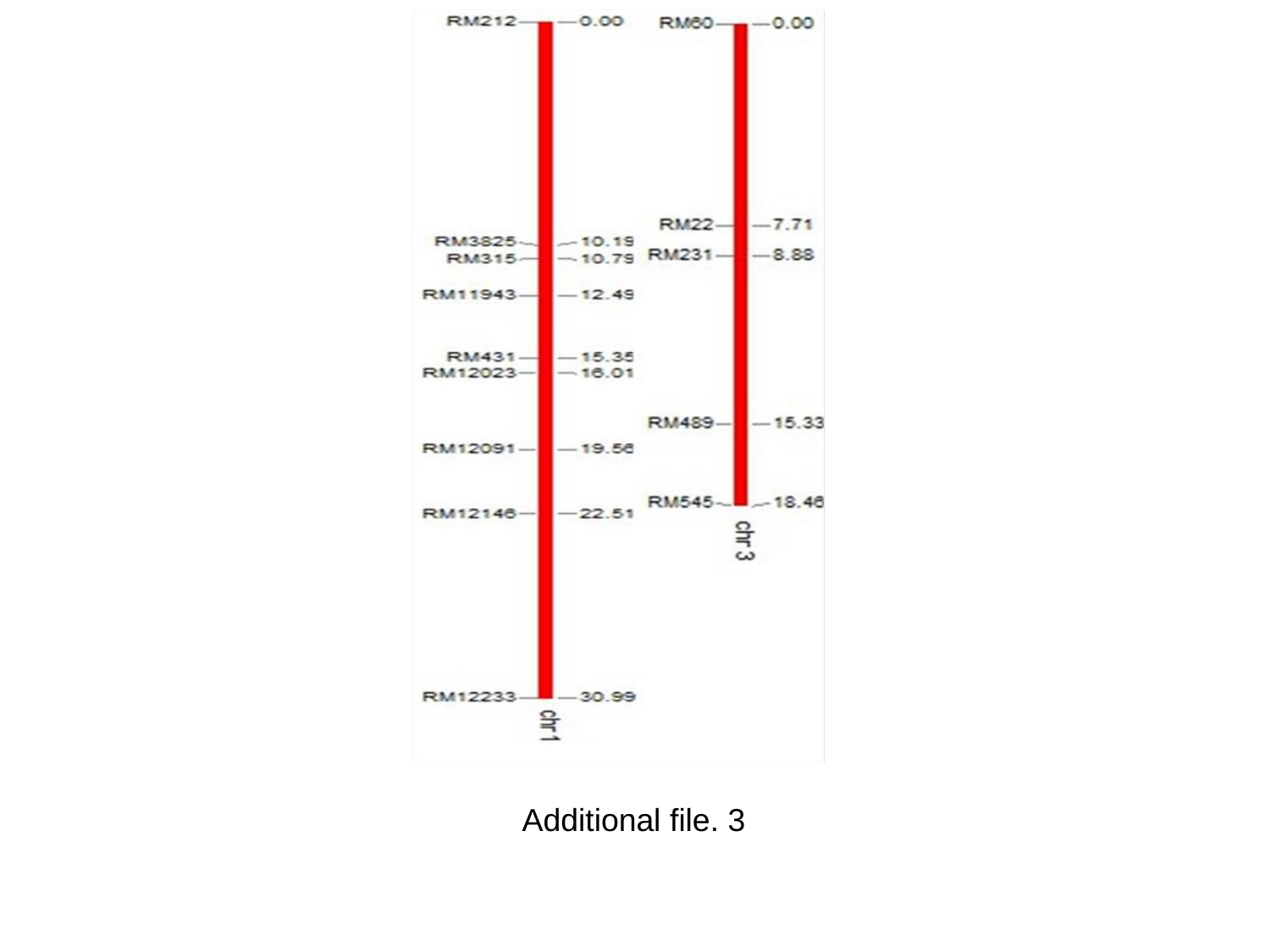

Additional file. 3

Supplement: Additional file 3 — Genetic map distances among markers on chromosomes 1 and 3 in N22/Swarna population. [file 1471-2156-12-89-S3.PPT]
